# Circulating Tumor Cell Clusters Are Cloaked with Platelets and Correlate with Poor Prognosis in Unresectable Pancreatic Cancer

**DOI:** 10.3390/cancers13215272

**Published:** 2021-10-20

**Authors:** Minji Lim, Suhyun Park, Hyoung-Oh Jeong, Sung Hee Park, Sumit Kumar, Aelee Jang, Semin Lee, Dong Uk Kim, Yoon-Kyoung Cho

**Affiliations:** 1 Center for Soft and Living Matter, Institute for Basic Science (IBS), Ulsan 44919, Korea; ming4279@gmail.com (M.L.); sumitwithchem@gmail.com (S.K.); 2 Department of Biomedical Engineering, Ulsan National Institute of Science and Technology (UNIST), Ulsan 44919, Korea; suhyunpark@unist.ac.kr (S.P.); hyoung-oh@unist.ac.kr (H.-O.J.); seminlee@unist.ac.kr (S.L.); 3 Department of Internal Medicine and Biomedical Research Institute, Pusan National University Hospital, 179, Gudeok-ro, Seo-Gu, Busan 49241, Korea; scaletlee@hanmail.net; 4 Department of Nursing, University of Ulsan, Ulsan 44610, Korea; aeleejang@ulsan.ac.kr

**Keywords:** circulating tumor cells, circulating tumor cell clusters, pancreatic cancer, platelets

## Abstract

**Simple Summary:**

Despite recent advances, some patients with pancreatic cancer are refractory to treatment and the disease rapidly progresses, resulting in early death. The potential prognostic value of circulating tumor cells (CTCs) has been demonstrated in other cancer types, but the clinical validity in pancreatic cancer remains elusive. Here, we show that CTC clusters, which show mesenchymal characteristics and platelet marker expression, are highly correlated with poor prognosis in patients with unresectable pancreatic cancer.

**Abstract:**

Circulating tumor cells (CTCs) are known to be heterogeneous and clustered with tumor-associated cells, such as macrophages, neutrophils, fibroblasts, and platelets. However, their molecular profile and clinical significance remain largely unknown. Thus, we aimed to perform a comprehensive gene expression analysis of single CTCs and CTC clusters in patients with pancreatic cancer and to identify their potential clinical relevance to provide personalized medicine. Epitope-independent, rapid (>3 mL of whole blood/min) isolation of single CTCs and CTC clusters was achieved from a prospective cohort of 16 patients with unresectable pancreatic cancer using a centrifugal microfluidic device. Forty-eight mRNA expressions of individual CTCs and CTC clusters were analyzed to identify pancreatic CTC phenotype. CTC clusters had a larger proportion of mesenchymal expression than single CTCs (*p* = 0.0004). The presence of CTC clusters positively correlated with poor prognosis (progression-free survival, *p* = 0.0159; overall survival, *p* = 0.0186). Furthermore, we found that most CTCs in these patients (90.7%) were cloaked with platelets and found the presence of a positive correlation between the increase in CTC clusters and rapid disease progression during follow-ups. Efficient CTC cluster isolation and analysis techniques will enhance the understanding of complex tumor metastasis processes and can facilitate personalized disease management.

## 1. Introduction

Pancreatic cancer is a leading cause of cancer mortality worldwide [1,2]. Pancreatic cancer showed a dismal prognosis with a 5-year survival rate of <9% because most patients were diagnosed in the late phase owing to rapid progression and early metastasis associated with the disease [3]. Although recent advances in therapeutic options such as the FOLFIRINOX regimen and gemcitabine with nab-paclitaxel showed survival benefits in patients with advanced pancreatic ductal adenocarcinoma (PDAC), some patients may be refractory to these chemotherapeutic agents, resulting in rapid disease progression and early death. Therefore, better predictive biomarkers are needed to select proper candidates for chemotherapy with unresectable pancreatic cancer [3].

Circulating tumor cells (CTCs) are good candidates for prognostic biomarkers since their evaluation is minimally invasive and therefore allows for more frequent monitoring of the real-time dynamics of cancer progression [2,3,4,5,6]. Despite the potential benefits [7,8,9,10], the clinical utility of CTCs in pancreatic cancer has not been fully elucidated, partly because of the intrinsic difficulties associated with the rareness and heterogeneity of CTCs. While many previous studies have used immunoaffinity-based CTC capture using anti-epithelial cell adhesion molecule (EpCAM) antibodies, emerging evidence suggests that CTCs undergo epithelial–mesenchymal transition (EMT) to enter the bloodstream and seeding distant organs. Therefore, it is essential to enrich CTCs independent of the surface markers and to characterize CTCs at a single-cell level to understand the phenotype heterogeneity and dynamics as well as their potential clinical relevance [2,6].

CTCs can migrate through the stroma and blood vessels not only as single cells but also as clusters, which are groups of two or more aggregated CTCs [10,11,12,13]. Several studies have suggested that CTC clusters might be related to a higher metastatic potential or poor prognosis. In the early 1970s, a greater association of CTC clusters with distal metastasis than of single CTCs was demonstrated through a series of preclinical studies [14,15]. The prognostic value of CTC clusters has been demonstrated in recent studies on patients with lung, breast, and prostate cancers [11,16,17,18]. In PDAC, CTC clusters (also circulating tumor microemboli) were shown to be an independent prognostic factor of progression-free survival (PFS) and overall survival (OS) [10].

Not only the enumeration of CTC clusters but also their molecular characterization is important for discovering their potential clinical implications. Molecular analysis of CTC clusters can provide additional insights into the mechanisms, including how CTC clusters can survive during circulation and generate distant metastases. A promising hypothesis is related to the non-tumorous components of CTC clusters, such as platelets, neutrophils, macrophages, and fibroblasts, which are expected to offer a survival advantage by shielding CTC clusters from shear forces, environmental or oxidative stresses, and immune assault [19,20,21,22,23,24]. However, to date, only a few studies have reported the molecular characterization of patient-derived PDAC CTC clusters and their clinical implications [25,26,27]. An in-depth understanding of the molecular characteristics of CTC clusters and their clinical significance is required to provide more effective precision medicine options.

Thus, we aimed to achieve a comprehensive phenotypic characterization of CTC clusters based on mRNA profiling and to demonstrate a correlation between the molecular phenotype of CTC clusters and clinical outcomes in patients with unresectable pancreatic cancer. We performed phenotypic characterization of CTCs based on mRNA profiling of individual CTCs or CTC clusters using a multiplex polymerase chain reaction chip capable of simultaneous analysis of 48 genes that are associated with EMT phenotypes, stemness, and the presence of platelets, neutrophils, macrophages, and fibroblasts.

## 2. Results

### 2.1. Patients’ Characteristics and CTC Enumeration

**Figure 1:**
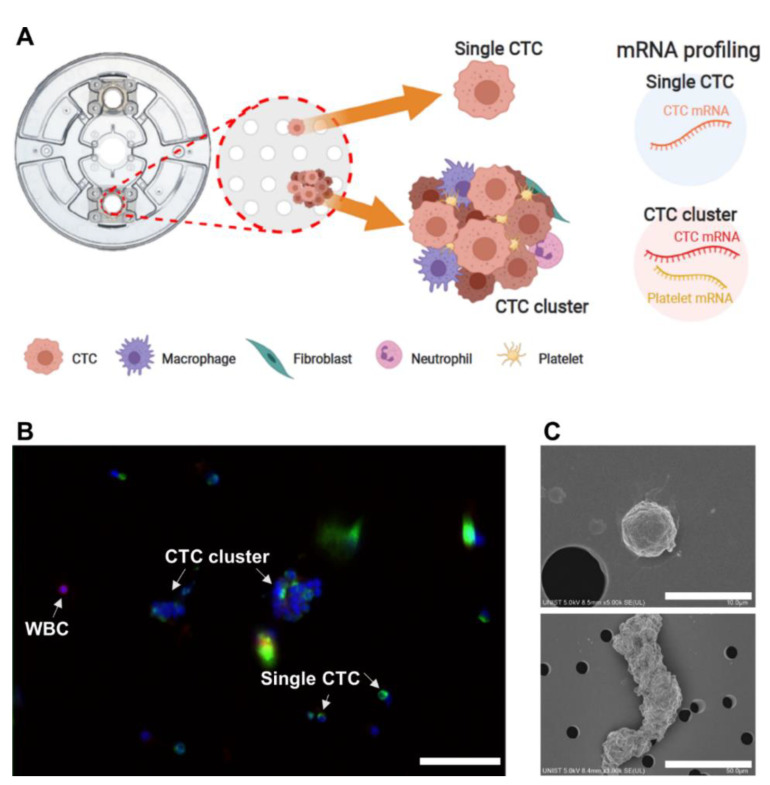
Scheme of circulating tumor cell (CTC) characterization. (**A**) Scheme of CTC characterization using mRNA profiling. (**B**) Immunofluorescence image of CTCs and white blood cell (WBC) (scale bar: 50 µm). (**C**) Scanning electron microscope images of single CTC and CTC cluster.

We employed a centrifugal microfluidic device, the FAST disc, to achieve label-free isolation of CTCs from whole blood samples without pretreatment [28,29]. Single CTCs showing CD45^−^ and DAPI^+^ expression and CTC clusters captured on the membrane of the FAST disc were picked without fixation and used for molecular analysis (Figure 1A). To investigate the heterogeneity of CTCs and their interaction with other cell types in the blood microenvironment, we analyzed genes associated with EMT, proliferation, and other types of cells, including platelets, macrophages, neutrophils, and fibroblasts (Table S1). In addition, we performed immunofluorescence staining (Figure 1B and Figure S1) and took SEM (Figure 1C and Figure S2) images to visualize CTCs and CTC clusters and to test conventional criteria for CTC identification, DAPI^+^, EpCAM/CK^+^, and CD45^−^.

Overall, 16 patients (mean age, 66.9 years; range, 51–80 years) with histologically proven PDAC between March 2018 and October 2019 were enrolled (Table S2). For all patients, the initial blood draw was collected in treatment-naïve status (baseline, V0). Six patients were followed up at an interval of 2–3 months with a blood draw. Eleven of the 16 patients (68.8%) were diagnosed with metastatic disease and the other five patients with locally advanced disease. The median follow-up time for all patients was 228 days (range, 45–381 days). Fourteen patients (87.5%) showed disease progression during a median follow-up of 129 days (range, 45–303 days). One patient died owing to sudden cardiac arrest. Three patients (S007, S018, and S025) died without any image follow-up within 60 days. These patients were defined as the rapid progression in metastasis (RP-M) group, and the other 13 patients (81.3%) who survived for more than 60 days were defined as the non-rapid progression in metastasis (non-RP-M) group. From the RP-M group, 9 single CTCs and 34 CTC clusters were detected; CTC clusters constituted 79.1% among the total of 43 CTCs, while 23 single CTCs and 11 CTC clusters were detected in the non-RP-M group.

At baseline, the detection rate of CTC clusters was much higher in pancreatic cancer patients with metastatic disease (5/11, 45.5%) than those with locally advanced disease (1/5, 20.0%). Among the five patients with metastatic pancreatic cancer with CTC clusters, three patients were classified into the RP-M group. The other six patients among the 11 patients with metastatic disease and without CTCs were classified into the non-RP-M group.

### 2.2. Molecular Characterization of Heterogeneity of Five Pancreatic Cancer Cell Lines

Before the analysis of patient-derived CTCs, we first tested five pancreatic cancer cell lines: BxPC-3, PANC-1, Capan-1, HPAC, and MIA PaCa-2. After FAST disc operation similar to the patients’ sample processing using blood samples spiked with cancer cells (~500 cells/3 mL), each live cell captured on the membrane (*n* = 5 per cell type) was collected using a single-cell manipulation technique and was subjected to mRNA expression analysis. The t-SNE analysis was able to recapitulate five different cell clusters corresponding to different cell lines (Figure S4A). In the correlation matrix plot showing Spearman’s correlation coefficient among 25 individual cells from the five different cell lines (Figure S4B), the mesenchymal cell lines (HPAC and MIA PaCa-2) showed a highly positive correlation with each other but showed a strong negative correlation with the other epithelial cell lines (BxPC-3, PANC-1, and Capan-1). Additionally, our mRNA expression-based single-cell scoring system [29] could clearly recapitulate well-known characteristics of each cell line (Figure S4C–F). The epithelial cell lines achieved significantly higher scores for epithelial markers, namely KRT 19, KRT7, and CDH1, with scores of 91.5 ± 7.0, 72.9 ± 21.4, and 78.8 ± 2.9 for BxPC-3, PANC-1, and Capan-1, respectively, and 13.9 ± 25.7 and 6.5 ± 1.2 for HPAC and MIA PaCa-2, respectively. In contrast, the mesenchymal scores related with mesenchymal markers (vimentin, SPARC, and SNAI1) were 0.5 ± 0.7, 8.5 ± 15.1, and 0.0 ± 0.0 for BxPC-3, PANC-1, and Capan-1, respectively, and 49.3 ± 41.4 and 50.8 ± 11.8 for HPAC and MIA PaCa-2, respectively. Moreover, the expression of stem-cell-like markers (CD44, NANOG, and PROM1) was slightly higher in the mesenchymal cell lines, with scores of 8.0 ± 6.4, 18.6 ± 9.0, and 21.2 ± 2.9 for BxPC-3, PANC-1, and Capan-1, respectively, and 36.8 ± 33.6 and 42.7 ± 12.6 for HPAC and MIA PaCa-2, respectively.

### 2.3. mRNA Expression Profiling of Patient-Derived Single CTCs and CTC Clusters

**Figure 2:**
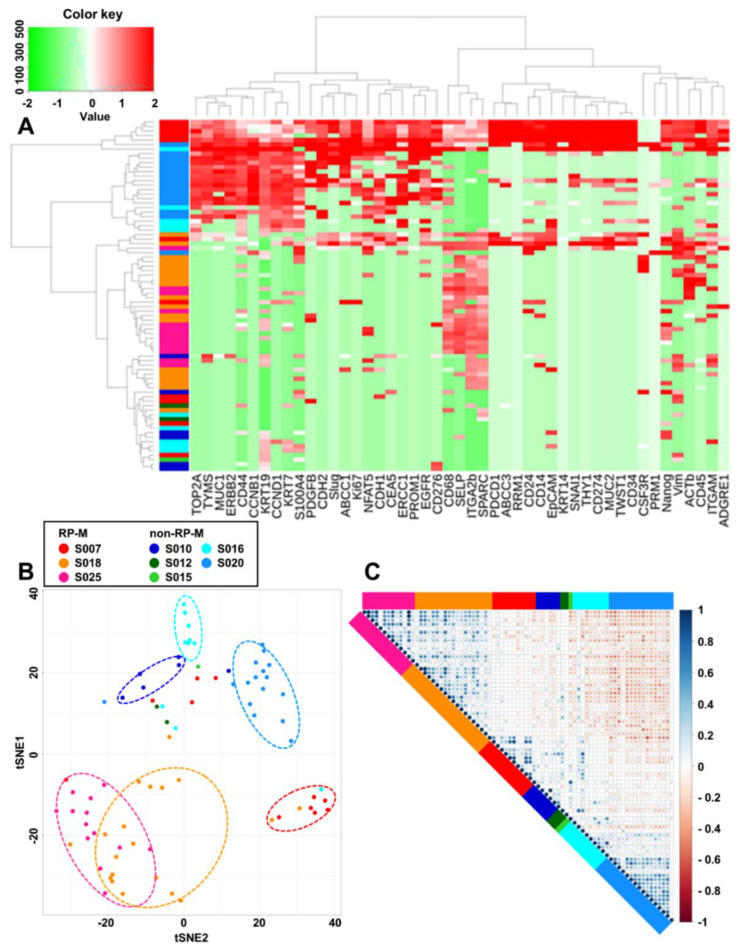
Clustering of patient-derived CTCs according to the sample source. (**A**) Heat map for hierarchical clustering of the differentially expressed gene expression profiles of a single cell, color-coded, from eight different patients. (**B**) Two-dimensional t-SNE analysis based on hierarchical clustering of eight pancreatic cancer patients in two groups according to the progression (rapid progression in metastasis (RP-M): progression in metastasis for <60 days; non-rapid progression in metastasis (non-RP-M): progression in metastasis for >60 days). (**C**) Correlation matrix plot for eight pancreatic cancer patients. t-SNE, t-distributed stochastic neighbor embedding.

mRNA profiling was performed on 77 CTCs (single CTCs (*n* = 32) and CTC clusters (*n* = 45)) from eight patients to analyze their molecular characteristics. Among them, 62 CTCs were obtained from the blood samples at baseline before chemotherapy, and the other 15 CTCs were obtained after chemotherapy. Unsupervised hierarchical clustering showed that the gene expression patterns were distinctive depending on the origin (Figure 2A). Despite the heterogeneity of individual CTCs and CTC clusters, CTCs from the same patient were clustered together as presented in the t-SNE plot (Figure 2B) and the correlation matrix (Figure 2C).

**Figure 3:**
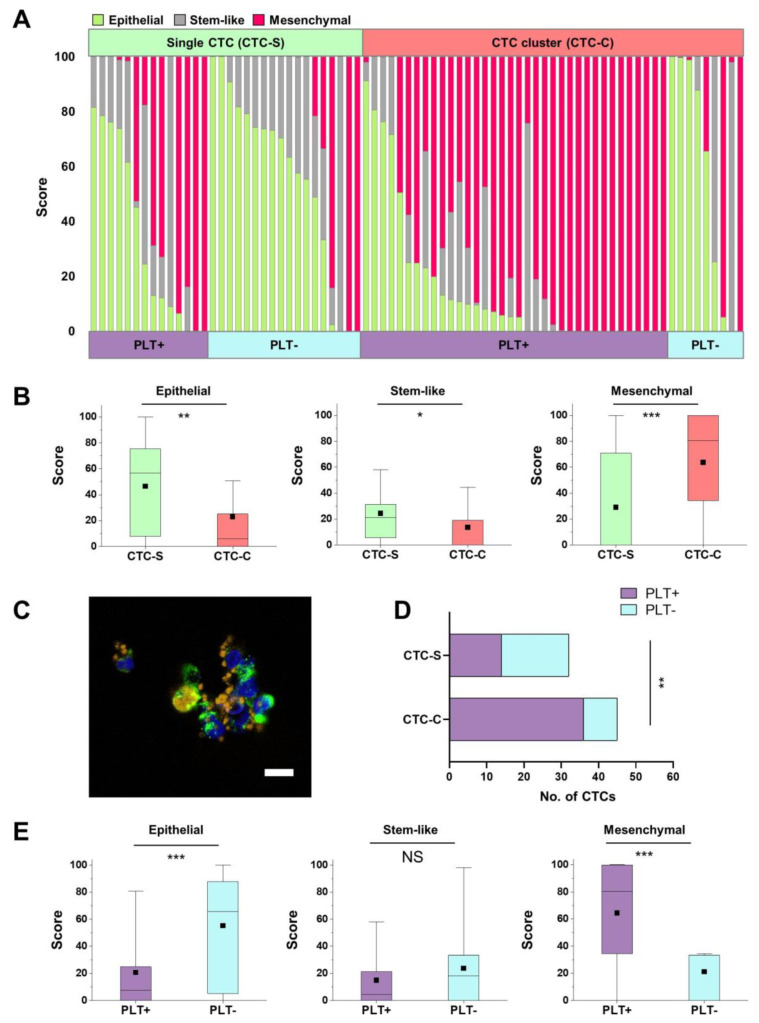
Circulating tumor cell (CTC) characterization according to the subtypes. (**A**) CTCs classified into three categories (epithelial, stem-like, and mesenchymal CTCs) according to their subtypes: (1) CTC-S (single CTC) or CTC-C (CTC cluster) and (2) PLT+ group (platelet marker-positive group) or PLT− group (platelet marker-negative group). (**B**) The score of the three categories between CTC-S and CTC-C. Box plots show 25th and 75th percentiles, with lines indicating the median value and black square dots indicating the mean value. All the differences for the three categories between the two groups are statistically significant (* *p* < 0.05, ** *p* < 0.01, *** *p* < 0.001). (**C**) Fluorescence images of CTC-S (left) and CTC-C (right) with P-selectin-stained platelets at 60× (scale bar: 10 µm). (**D**) Fisher’s exact test based on the analysis of contingency tables between CTC-S and CTC-C according to the platelet marker expression (*p* = 0.0015). (**E**) The score for the three categories between the PLT+ group and the PLT− group. Box plots show 25th and 75th percentiles, with lines indicating the median value and black square dots indicating the mean value. The differences between the two groups for epithelial and mesenchymal scores are statistically significant (*** *p* < 0.001; NS: non-significant).

Furthermore, very distinctive differences in mRNA expression patterns between 32 single CTCs and 45 CTC clusters were observed (Figure 3A). The epithelial score was higher for single CTCs (46.5 ± 34.6 and 23.0 ± 33.4 for single CTCs and CTC clusters, respectively; *p =* 0.004), while the mesenchymal score was distinctively higher for CTC clusters (29.1 ± 39.7 and 63.6 ± 39.4 for single CTCs and CTC clusters, respectively; *p =* 0.0004). The stemness marker score was slightly higher for single CTCs than for CTC clusters (24.4 ± 23.6 and 13.4 ± 22.7 for single CTCs and CTC clusters, respectively; *p =* 0.046) (Table S3 and Figure 3B).

### 2.4. Platelet-Associated Genes in Single CTCs and CTC Clusters

Consistent with previous studies reporting pro-tumorigenic roles of platelets in tumor metastasis [30,31,32,33,34,35,36,37,38,39], we could identify platelet-covered CTCs in the fluorescence and SEM images (Figure 3C, Figures S2C and S3). Platelet markers were present in both single CTCs and CTC clusters but were higher in CTC clusters (43.75% vs. 80%) (Figure 3A,D).

Next, we characterized the molecular makeup of individual CTCs depending upon the presence of platelet markers. CTCs were divided into two groups according to the presence of platelet marker expression: PLT+ group (at least one among three platelet markers (ITGA2b, SELP, and PDGFb)) and PLT− group (no platelet marker expression). Among the CTCs in the PLT+ group, 72.0% were CTC clusters and 28.0% were single CTCs (Figure 3A). The PLT+ group showed significantly lower epithelial scores (20.7 ± 28.7 vs. 55.1 ± 37.0; *p* = 0.00003) and significantly higher mesenchymal scores (64.4 ± 38.2 vs. 21.2 ± 36.9; *p* = 0.00001) than the PLT− group. Among the four different categories of CTCs, depending upon the presence of platelet markers and cluster form, the PLT+/CTC cluster group showed the highest mesenchymal scores (73.0%), while the epithelial scores were highest in the PLT−/single CTC group (55.8%) (Figure 3E and Table S4).

### 2.5. Genes Associated with the Alliance of CTCs to Evade the Immune System

Approximately 80% of CTC clusters were associated with platelets, and we also investigated other types of blood cells in CTC clusters. Two immune cells (macrophages and neutrophils) and one stromal cell type (fibroblasts), which are known to be associated with the tumor microenvironment, were analyzed as well. CTC clusters showing positive expression of at least one macrophage-related marker (CD68, CD14, or ADGRE1) were defined as Mac+ CTC clusters. Similarly, CTC clusters with at least one neutrophil-related marker (CD45, CSF3R, or ITGAM) were defined as Neu+ CTC clusters. CTC clusters with fibroblast-related markers were defined as Fib+ CTC clusters.

Overall, 39 of the 45 CTC clusters (86.7%) expressed at least one stromal or immune cell marker. Among the 39 CTC clusters with stromal or immune cells, 32 CTC clusters (82.1%) expressed platelet markers (Figure S5 and Table S5). Approximately 92.6% of Mac+ CTC clusters, 88.5% of Neu+ CTC clusters, and 75.0% of Fib+ CTC clusters had platelet marker expression, which may imply the role of platelets in the adherence of cells to form CTC clusters (Table S5).

### 2.6. CTC Clusters Are Cloaked with Platelets and Correlate with Poor Prognosis in Patients with Pancreatic Cancer

**Figure 4:**
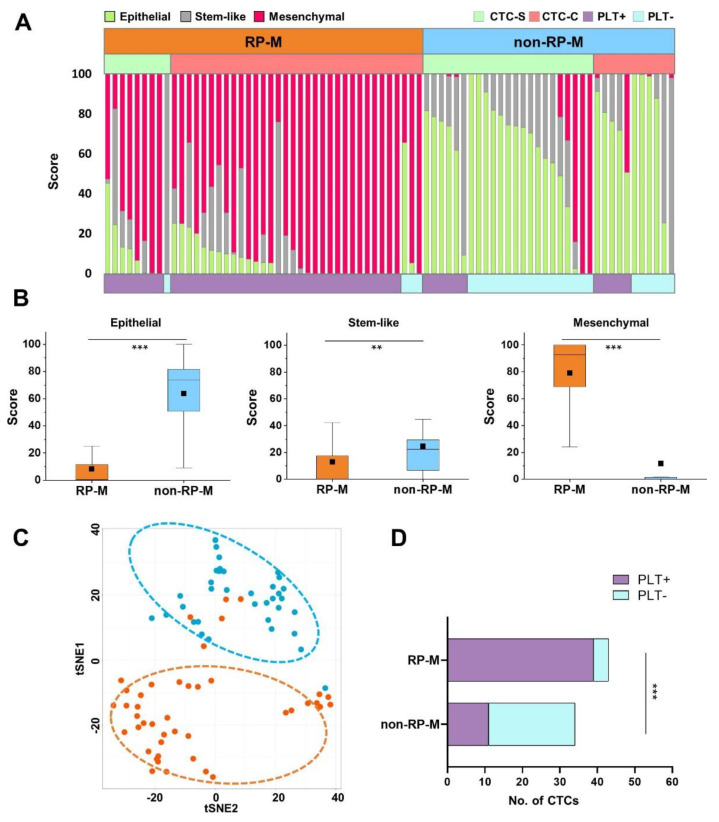
Circulating tumor cell (CTC) characterization according to the progression. (**A**) CTC classification into three categories between the rapid progression in metastasis (RP-M) group and the non-rapid progression in metastasis (non-RP-M) group according to subtypes: (1) CTC-S (single CTC) or CTC-C (CTC cluster) and (2) PLT+ group (platelet marker-positive group) or PLT− group (platelet marker-negative group). (**B**) The score of the three categories between the RP-M group and non-RP-M group (** *p* < 0.01, *** *p* < 0.001). (**C**) Two-dimensional t-SNE analysis based on hierarchical clustering between the RP-M group and non-RP-M group. (**D**) Fisher’s exact test based on the analysis of contingency tables between the RP-M and non-RP-M groups according to the platelet marker expression (*p* < 0.0001). t-SNE, t-distributed stochastic neighbor embedding.

We further analyzed the CTC characteristics based on the clinical outcomes. Among the eight patients whose CTCs were detected, clinical assessments were divided into RP-M and non-RP-M groups. As shown in the X-ray and CT images in Figure S6, three patients (S007, S018, and S025) in the RP-M group showed rapid progression of metastasis or an increased tumor mass. Of the 77 CTCs detected in eight patients, 43 CTCs (56%) were from those three patients in the RP-M group. Notably, 76% of CTC clusters (34/45) were detected in those three patients in the RP-M group (Figure 4). Single CTCs and CTC clusters accounted for 20.9% (9/43) and 79.1% (34/43), respectively, in the RP-M group. It is important to note that not only CTC clusters (82.6%) but also single CTCs (65.4%) in the RP-M group showed high mesenchymal scores (Table S6). Figure 4A clearly demonstrates that CTCs in the RP-M group showed significantly higher mesenchymal characteristics than those in the non-RP-M group (79.0 ± 26.4 vs. 11.7 ± 27.9; *p* < 0.001, Figure 4B). Despite the heterogeneity of individual CTCs, the t-SNE analysis clearly identified CTCs based on the clinical outcomes (RP-M group vs. non-RP-M group) (Figure 4C). We further analyzed the association of platelet marker expression and clinical outcome. Most CTCs in the RP-M group (90.7%) had platelet marker expression, while more than half of the CTCs in the non-RP-M group (67.7%) had no platelet marker expression (Figure 4D).

**Figure 5:**
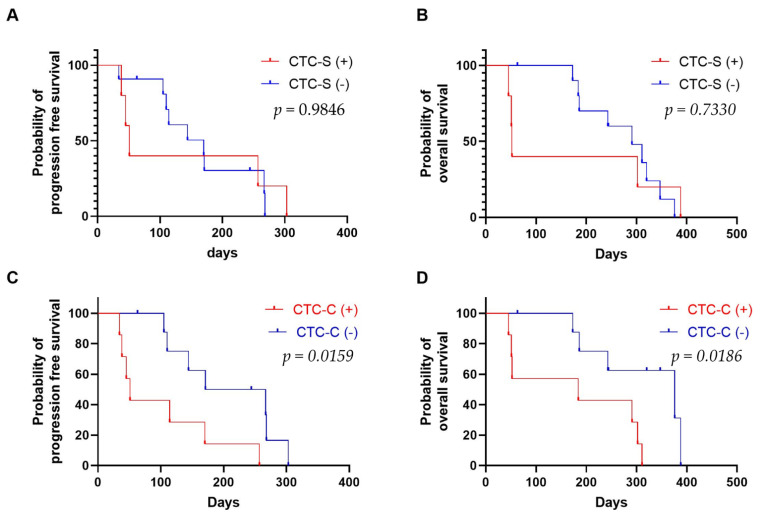
Survival analysis. (**A**) Progression-free survival and (**B**) overall survival according to the presence of CTC-S (single CTCs) among the 16 pancreatic cancer patients. (**C**) Progression-free survival and (**D**) overall survival according to the presence of CTC-C (CTC clusters) among the 16 pancreatic cancer patients. CTC-S (+) and CTC-C (+) are for the patients who have one or more single CTCs and CTC clusters, respectively. CTC-S (-) and CTC-C (-) are for the patients who do not have single CTCs and CTC clusters, respectively.

The presence of single CTCs did not correlate with survival (PFS, *p* = 0.9846; OS, *p* = 0.7330) (Figure 5A,B). However, the presence of CTC clusters was strongly related to PFS and OS (PFS, *p* = 0.0159; OS, *p* = 0.0186) (Figure 5C,D).

**Figure 6:**
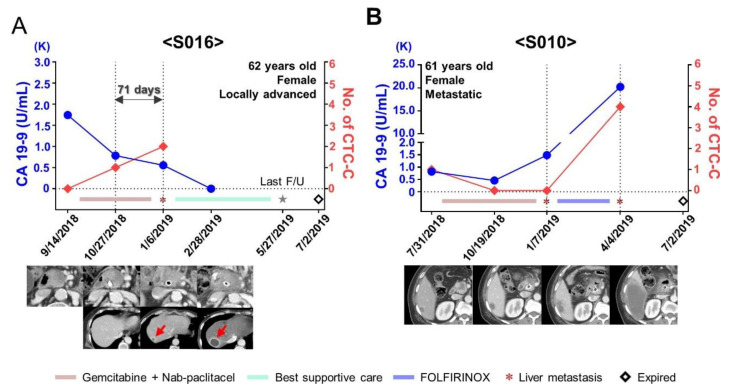
Longitudinal follow-up of unresectable pancreatic cancer patients. (**A**) Changes in the level of carbohydrate antigen 19-9 (CA 19-9) and the number of CTC-C (circulating tumor cell (CTC) clusters) in patient S016 during the follow-up. An increase in the number of CTC-C was found 71 days before the evidence of progression with computed tomography. (**B**) Changes in the level of CA 19-9 and the number of CTC-C in patient S010 during the follow-up. S010 had the presence of CTC-C accompanying liver metastasis at baseline treatment-naïve status. CTC-C were not detected after starting chemotherapy; however, the number of CTC-C surged when the liver mass suddenly increased.

There were six patients who underwent chemotherapy and had more than one blood draw during follow-ups. In two patients with locally advanced disease (S013 and S019), no CTCs were detected at baseline and during follow-up and no progression was observed. Two patients with locally advanced disease (S012 and S020) showed relatively long PFS based on several single CTCs at baseline and a decreased number of CTCs during follow-ups. Furthermore, two more patients (S016 and S010) were available to observe the changes in CTC cluster counts and the changes in carbohydrate antigen 19-9 (CA 19-9) level because of their sequential blood sampling (>3 time points, including baseline). One patient with locally advanced disease (S016) showed an increased number of CTCs during the first follow-up even though she did not have CTCs at baseline; moreover, she did not present any evidence of progression based on the CT finding and CA 19-9 level. However, the patient showed new liver metastasis with more increased CTCs. We observed an increased number of CTC clusters 71 days before the evidence of progression using CT (Figure 6A). The other patient (S010) with a metastatic liver mass showed CTC clusters at baseline. Although the size of the liver mass increased by <20%, the CA 19-9 level decreased after starting chemotherapy. Finally, the liver mass showed progression according to the Response Evaluation Criteria in Solid Tumors at six months after starting chemotherapy. The patient did not show CTC clusters after starting chemotherapy; however, when she had a sudden increase in the liver mass, her CTC cluster count also surged (Figure 6B).

## 3. Discussion

Overall, 58.4% (45/77) of the total CTCs analyzed in our study were CTC clusters; 80.0% (40/50) of CTCs isolated from patients with metastasis were CTC clusters, while 81.5% (22/27) of CTCs from patients with locally advanced disease were single CTCs. Remarkably, 85% (34/40) of CTC clusters in patients with metastasis were from the RP-M group. The presence of CTC clusters is known to be a prognostic factor for PDAC for both PFS and OS. While our findings support the findings of previous studies suggesting a higher metastatic potential of CTC clusters [10,20], we have further characterized the molecular phenotype of individual CTCs and CTC clusters.

Based on the analysis of the heterogeneous population of PDAC CTC clusters in terms of epithelial vs. mesenchymal phenotype, mesenchymal markers were the highest in CTC clusters (63.60%) and in the RP-M group (78.98%), which confirms the findings of previous studies reporting the mesenchymal characteristics of CTC clusters. It is worth mentioning that the fact that mesenchymal markers are highest in clusters may not necessarily mean that the tumor cells inside the clusters are mesenchymal, but the clusters are heterotypic and contain accessory cells from blood lineage that do express mesenchymal markers. In addition to the well-known mesenchymal markers vimentin and SNAI1 [40], SPARC was also included because of the highly positive correlation with mesenchymal markers such as vimentin, ZEB1, N-cadherin, and Twist and the highly negative correlation with the epithelial marker E-cadherin [26,41]. Although it is not a major fraction, 22.2% (10/45) of CTC clusters were of epithelial type, which were assumed to be disseminated from primary cancer via non-EMT-mediated invasion, thus retaining their epithelial characteristics during circulation [11,42]. In our study, only one among the total 10 epithelial CTC clusters originated from a patient in the RP-M group.

Regarding the presence of other cells in CTC clusters traveling together in the bloodstream, we observed platelet markers in 80% (36/45) of the total CTC clusters, and 43.8% of single CTCs (14/32) were platelet-associated. Intriguingly, 91% (31/34) of CTC clusters detected in the RP-M group had platelet marker expression. Moreover, CTC clusters showed various combinations of RNA expressions related to immune cells. Among the 45 CTC clusters, 60% had a positive expression for macrophage markers, 57.78% showed neutrophil marker expression, and 44.44% had fibroblast marker expression. Remarkably, macrophage- (92.6%), neutrophil- (88.5%), and fibroblast-associated (75.0%) CTC clusters also showed positive platelet marker expression. It is implied that platelets may have influenced the recruitment of immune and stromal cells to form CTC clusters.

There is mounting evidence suggesting the important role of platelets in tumor cell circulation in blood and cancer metastasis [30,31,32,33,34,35,36,37,38,39]. Platelets form aggregates with tumor cells by binding to tumor-derived tissue factor and thrombin, which can protect CTC clusters from shear stress or immune attacks in the bloodstream [30,31,32,33,34,35]. They also promote metastasis by helping the adhesion of CTCs to endothelial cells of the vessel wall and form early metastatic niches [30,31,32,33,34,35]. Platelets are known to have direct interaction with immune cells and modulate the immune response during inflammation [33]. The interplay of platelets with tumor cells has been utilized both in cancer diagnostics and advanced therapies [36,37,38,39]. Moreover, emerging evidence supports the heterogeneity in the complex composition of CTC clusters [43]. For example, studies having an in-depth understanding of the roles of tumor-associated macrophages [44,45], neutrophils [23], and carcinoma-associated fibroblasts [46] in promoting tumor metastasis have been reported.

Although the importance of platelet-covered CTC clusters is recognized, few studies have reported the specific molecular characterization and clinical significance of platelets in tumor progression. Jiang et al. observed platelet-covered CTCs from patients with lung and breast cancers using a platelet-specific CD41 antibody-coated microfluidic chip [32]. Aceto et al. detected platelet markers in both single CTCs and CTC clusters isolated from breast cancer patients [11]. Beck et al. revealed that the expression of platelet-associated genes in CTCs and cell-free RNA was associated with patients’ survival [30]. In our study, we could isolate single CTCs and CTC clusters using the FAST disc from a prospective cohort of PDAC patients and demonstrate the prognostic value of platelet-covered CTCs using a comprehensive single-cell mRNA expression analysis.

Although the importance of CTC clusters in cancer metastasis and disease outcome has been highly recognized [10,11,13], studies on the molecular characteristics of PDAC CTCs and CTC clusters at a single-cell level are limited [25,26,27], partly because of the difficulties associated with the rare, fragile, and heterogeneous nature of CTCs. In our study, single-cell mRNA expression analysis of CTCs and CTC clusters was performed using label-free isolation of CTCs from the whole blood of patients using the FAST disc [28]. Since CTCs may undergo phenotype change through EMT and hide in stealth mode for immune escape, a surface-marker-independent CTC isolation approach was chosen. The gentle spinning of the disc at FAST mode (600 rpm, <20 G-force) [28] allowed ultrafast (<20 s for 3 mL of whole-blood filtration) CTC isolation with a reduced pressure drop (~1 kPa); CTCs and CTC clusters were not stuck inside of the pore but sat on top of the membrane (Figure S2). Thus, it was easy to pick single cells and intact clusters and use live cells directly, without fixing, for more efficient single-cell gene expression analysis [29].

There are some important limitations to our study. First, the number of samples was small. A further study in a larger cohort is needed to explore clinical implications. Second, although we used an epitope-independent approach to isolate CTCs, small-sized cells were not captured on the filter membrane, and therefore, the analysis could be biased to the group of cells > 8 µm. Third, despite the fast collection of live CTCs from whole blood, several CTCs (15 samples among a total of 92 CTCs, 16%) had low-quality RNA and were thus excluded from the analysis. While the FAST disc has a lower failure rate than other live CTC capture methods (~41%) [25,26,47], it needs to be improved in future work. It is worth noting that all low-quality RNA samples belonged to patients in the non-RP-M group with only one or two isolated CTCs. Although all the blood samples in our study were processed within 6 h after blood withdrawal, reducing the sample transfer time before starting the enrichment process may help. Fourth, the current gene expression panel was designed to differentiate between epithelial and mesenchymal CTCs and to identify the presence of other blood cells in alliance with CTCs. Future studies may include more markers associated with drug resistance and disease progression and preferably RNA sequencing of CTC clusters dissociated into individual cells to have a broader and single-cell-level understanding of CTC clusters. Additionally, it would be valuable to have a molecular analysis of CTCs in early-stage cancer samples to determine whether a specific category of CTC subpopulation has prognostic value.

## 4. Materials and Methods

### 4.1. Study Design and Clinical Sample Collection

This is a prospective cohort study that recruited 16 participants in a single tertiary referral center in South Korea. Inclusion criteria were (1) age ≥ 50 years; (2) locally advanced or metastatic pancreatic cancer diagnosed using ultrasound, computed tomography (CT), and magnetic resonance images; and (3) histologically confirmed as PDAC. Blood draws were collected at baseline and during image workup after treatment every 2–3 months. Informed consent was obtained from all patients. The ethics committee of the internal review board of Pusan National University Hospital approved the study (H-H-1801-020-062). The clinical research information service approved the study (KCT0003511).

### 4.2. Label-Free Isolation of CTCs Using a Fluid-Assisted Separation Technology (FAST) Disc

The blood samples were collected in K2-EDTA tubes after discarding the first 1~2 mL of blood to avoid contamination with skin epithelial cells [48]. We used a commercialized version of the lab-on-a-disc equipped with FAST [28] on the CD-PRIME™ system (Clinomics, Ulsan, Korea) to isolate intact CTCs from whole blood. The CD-CTC™ Duo is a centrifugal microfluidic device for label-free, size-based CTC isolation that can be operated using a standalone spinning system, CD-OPR-1000™. Rapid (>3 mL of whole blood/min) and clog-free isolation of CTCs from the whole blood without any pretreatment steps was enabled using tangential flow filtration on a FAST disc [28,29].

### 4.3. Imaging of CTCs by Immunofluorescence Staining

In addition to the mRNA-expression-based CTC analysis, we also performed conventional immunofluorescence staining and image-based CTC enumeration. The immunostaining process was also performed on the disc. Subsequent to fixation of the captured cells, the cells were permeabilized with 0.1% Triton X-100 in PBS for 5 min and then washed with PBS. Then, the blocking step with 20 μg/mL of IgG was followed by staining with several antibodies. To stain the white blood cells, anti-CD45-conjugated PE/Alexa Fluor 610 (H130, Life Technologies, Carlsbad, CA, USA) was injected, incubated for 20 min, and then washed with 0.01% Tween 20 in PBS. Next, the CTCs were stained with a mixture of anti-cytokeratin-conjugated FITC (CAM5.2, BD, San Jose, CA, USA), anti-pan-cytokeratin-conjugated Alexa 488 (AE1/AE3, eBioscience, San Diego, CA, USA), and anti-EpCAM-conjugated FITC (9C4, BioLegend, San Diego, CA, USA). A mixture of these antibodies was introduced to the filter, incubated for 20 min, and then washed with 0.01% Tween 20 in PBS. Finally, the nuclei in the cells were stained with 4,6-diamidino-2-phenylindole (DAPI) [28]. Cells that were CK^+^/EpCAM^+^, CD45^−^, DAPI^+^, and morphologically intact were identified as CTCs, while CD45^+^ and DAPI^+^ cells were identified as WBCs. To confirm the presence of platelets in CTC clusters, anti-CD62P-conjugated APC (AK4, BioLegend, San Diego, CA, USA) was added for staining p-selectin of platelets. Stained cells were automatically imaged through a BioView workstation (BioView, Inc., Rehovot, Israel) for 10× images. A Nikon AR1 confocal microscope was used to obtain 60× fluorescence images.

### 4.4. Scanning Electron Microscope (SEM) Images of CTCs

To obtain SEM images, fixed cells were sequentially incubated in 5%, 10%, 20%, 40%, 60%, 80%, and 100% acetone for 20 min each followed by 2 h air-drying in between. Dehydrated cells were Au-sputtered and imaged using a Hitachi S-4800 Cold FE-SEM (Hitachi High-Technologies, Tokyo, Japan). All images were obtained under identical conditions at 5.00 kV accelerating voltage.

### 4.5. Cell Culture

Gene expression was validated using five types of pancreatic cancer cell lines, namely BxPC-3, Capan-1, PANC-1, HPAC, and MIA PaCa-2, purchased from the American Type Culture Collection (ATCC, Manassas, VA, USA). HPAC, PANC-1, and MIA PaCa-2 were cultured in DMEM containing 10% FBS and 1× antibiotic-antimycotic solution. BxPC-3 and Capan-1 were cultured in RPMI medium with 10% FBS and 1× antibiotic-antimycotic solution. All cells were cultured under 5% CO_2_ at 37 °C.

### 4.6. Single-Cell Isolation and Gene Expression Analysis

Individual CTCs showing CD45^−^ and DAPI^+^ expression and CTC clusters captured on the membrane were targeted for single-cell isolation using CellCelector™ (ALS, Jena, Germany) and subject to mRNA profiling. A CTC cluster composed of two or more CTCs (Figure 1B, Figures S1 and S3) was treated as one sample without dissociation. Single-cell cDNA was prepared using a Single Cell-to-Ct kit (Life Technologies, New York, NY, USA) and a pre-amplified specific target for gene expression analysis (Life Technologies, NY, USA). Quantitative reverse-transcription polymerase chain reaction was performed with the BioMark HD real-time PCR system (Fluidigm, South San Francisco, CA, USA). The list of genes used in our study is summarized in Supplementary Table S1.

### 4.7. Comprehensive Characterization of Single CTCs and CTC Clusters

Utilizing mRNA expression data from single CTCs and CTC clusters, we characterized CTCs into three categories: epithelial, stem-cell-like, and mesenchymal CTCs. Similar to our previous study [29], KRT7, KRT19, and CDH1 were used as characteristic markers for epithelial CTCs; CD44, NANOG, and PROM1 were used as markers for stem-cell-like CTCs; and vimentin, SPARC, and SNAI1 were used as markers for mesenchymal CTCs. mRNA expression of single CTCs and CTC clusters was normalized to that of GAPDH and calculated as the difference between the target gene Ct and GAPDH Ct values (2^−ΔCt^). Scores according to each type were defined as the sum of 2^−ΔCt^ values across the markers of each type. Finally, the total of scores from the three types was converted into a percentage. Additionally, platelet markers such as ITGA2b, SELP, and PDGFb were evaluated for the characterization of single CTCs and CTC clusters isolated from patients’ blood samples. CTC clusters were further analyzed for macrophage, neutrophil, and fibroblast markers. We used CD68, CD14, and ADGRE1 as macrophage markers; CD45, CSF3R, and ITGAM as neutrophil markers; and S100A4 and THY1 as fibroblast markers.

### 4.8. Assessment of Progression-Free Survival and Overall Survival

PFS and OS were evaluated for patients with CTC clusters. If an outcome was not reached, the time variables were censored at the last follow-up. Kaplan–Meier plots and the log-rank test were used to illustrate and compare survival between the subgroups. Survival analysis of variables measured at treatment-naïve baseline was performed. Univariate and multivariate hazard ratios for selected potential predictors of PFS and OS were determined using the Cox proportional hazards regression.

### 4.9. Statistical Analysis

Undetected genes were assigned a Ct value of 999, which were imputed using the highest Ct value observed for a given gene plus a value of 1 to provide balanced weights to missing data. All imputed Ct values in our statistical analyses were converted to a Z-score to provide the same weights [26]. We conducted unsupervised hierarchical clustering and t-distributed stochastic neighbor embedding (t-SNE) to explore associations among sample groups. Unsupervised hierarchical clustering and its heat map visualization were performed using the heatmap.2 function of the gplots package and t-SNE analysis was conducted using the Rtsne package for R (version 3.4.0, R Foundation for Statistical Computing, Vienna, Austria, https://www.R-project.org/, accessed on 21 April 2017).

We used the corrplot package to visualize the correlation matrix, and *p*-values were calculated using the cor.mtest function in R. The matrix shows pairwise Spearman rank correlations between the expression levels of indicated mRNAs in cells. The correlation matrix plots of correlations between the different expression levels of mRNAs measured were constructed with the corrplot function supplied with the corrplot package in R; the cor function was used to compute correlations. The correlation matrix was computed separately for patient-derived CTCs from each patient with Spearman rank correlations. Associated *p*-values were computed using the cor.mtest function in R. The Bonferroni correction of *p*-values was performed to adjust for multiple testing in the rank correlation matrix.

## 5. Conclusions

In conclusion, our study showed that CTC clusters, which showed mesenchymal characteristics and platelet marker expression, were highly associated with poor prognosis, including early death owing to rapid progression in metastasis. Furthermore, we confirmed the relationship between the increase in CTC clusters and rapid disease progression during follow-ups. Although further studies are needed to ascertain the clinical utility, these initial results suggest the potential role of CTC clusters in the progression of cancer and personalized medicine enabled using liquid biopsy.

## Data Availability

The data presented in this study are available on request from the corresponding author.

## Supplementary Materials

The following are available online at https://www.mdpi.com/article/10.3390/cancers13215272/s1, Figure S1: Fluorescence images at different channels of isolated CTCs and white blood cells shown in Figure 1B, Figure S2: Scanning electron microscope (SEM) images of single circulating tumor cell (CTC) and CTC cluster with platelets, Figure S3: Fluorescence images of circulating tumor cell (CTC) clusters with platelets, Figure S4: Characterization of pancreatic cancer cell lines using mRNA profiling, Figure S5: Investigation of heterogeneous components in circulating tumor cell (CTC) clusters, Figure S6: Serial chest X-ray images and computed tomography (CT) images of patients in the rapid progression in metastasis (RP-M) group, Table S1: Marker list, Table S2: Patient information, Table S3: Circulating tumor cell (CTC) classification between single CTCs and CTC clusters, Table S4: Circulating tumor cell (CTC) classification between single CTCs and CTC clusters according to the expression of platelet markers, Table S5: Characterization of circulating tumor cell (CTC) clusters according to the stromal and immune cell marker expressions, Table S6: Portion of the cases for the comparison between RP-M group and non-RP-M group.
