# Supplementary material for: Circulating Tumor Cell Clusters Are Cloaked with Platelets and Correlate with Poor Prognosis in Unresectable Pancreatic Cancer
Source: Cancers (Basel). 2021 Oct 20;13(21):5272. doi: 10.3390/cancers13215272 (PMC8582483; doi:10.3390/cancers13215272)
Supplement: Supplementary file 1 [file cancers-13-05272-s001.zip › cancers-1390607-supplementary_new.pdf]

Cancers **2021**, 13, 5272

Supplementary Materials: Circulating Tumor Cell Clusters Are Cloaked with Platelets and Correlate with Poor Prognosis in Unresectable Pancreatic Cancer

Minji Lim, Suhyun Park, Hyoung-Oh Jeong, Sung Hee Park, Sumit Kumar, Aelee Jang, Semin Lee, Dong Uk Kim and Yoon-Kyoung Cho

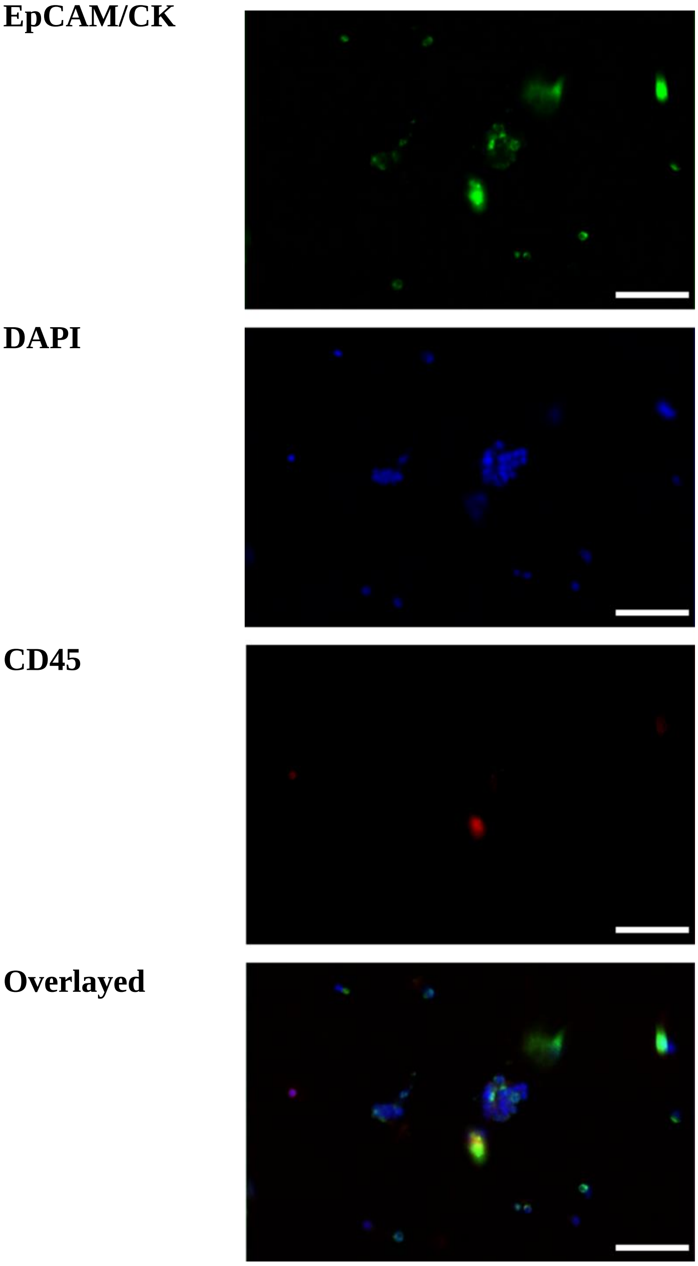

Figure S1. Fluorescence images at different channel of isolated CTCs and white blood cells shown in Figure 1B (scale bar: 50 μm).

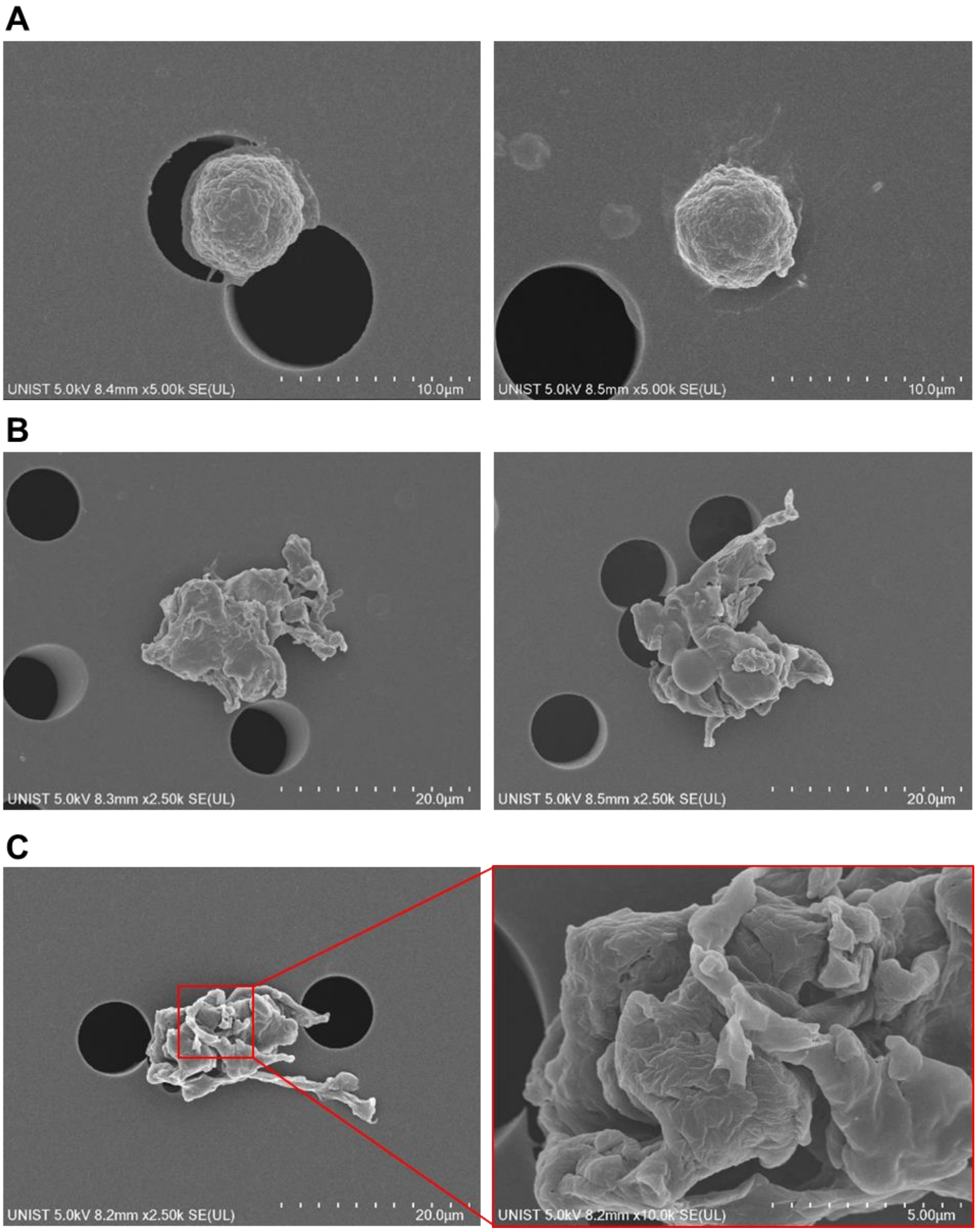

Figure S2. Scanning electron microscope (SEM) images of single circulating tumor cell (CTC) and CTC cluster with platelets. (**A**) SEM images of intact single CTCs from clinical samples. Single CTCs were found lying on the pore. (**B**) SEM images of CTC clusters. (**C**) Magnified SEM image of a platelet-covered CTC cluster.

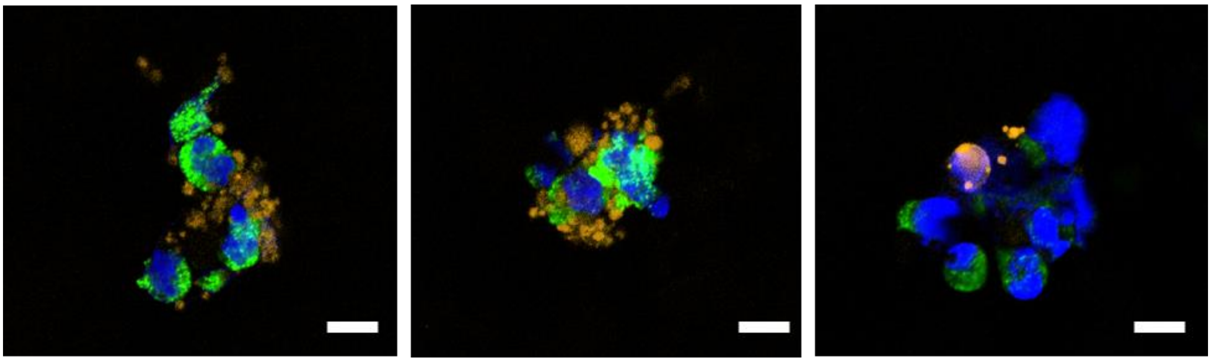

Figure S3. Fluorescence images of circulating tumor cell (CTC) clusters with platelets. Fluorescence images of CTC clusters with P-selectin-stained platelets at 60× (scale bar: 10 μm).

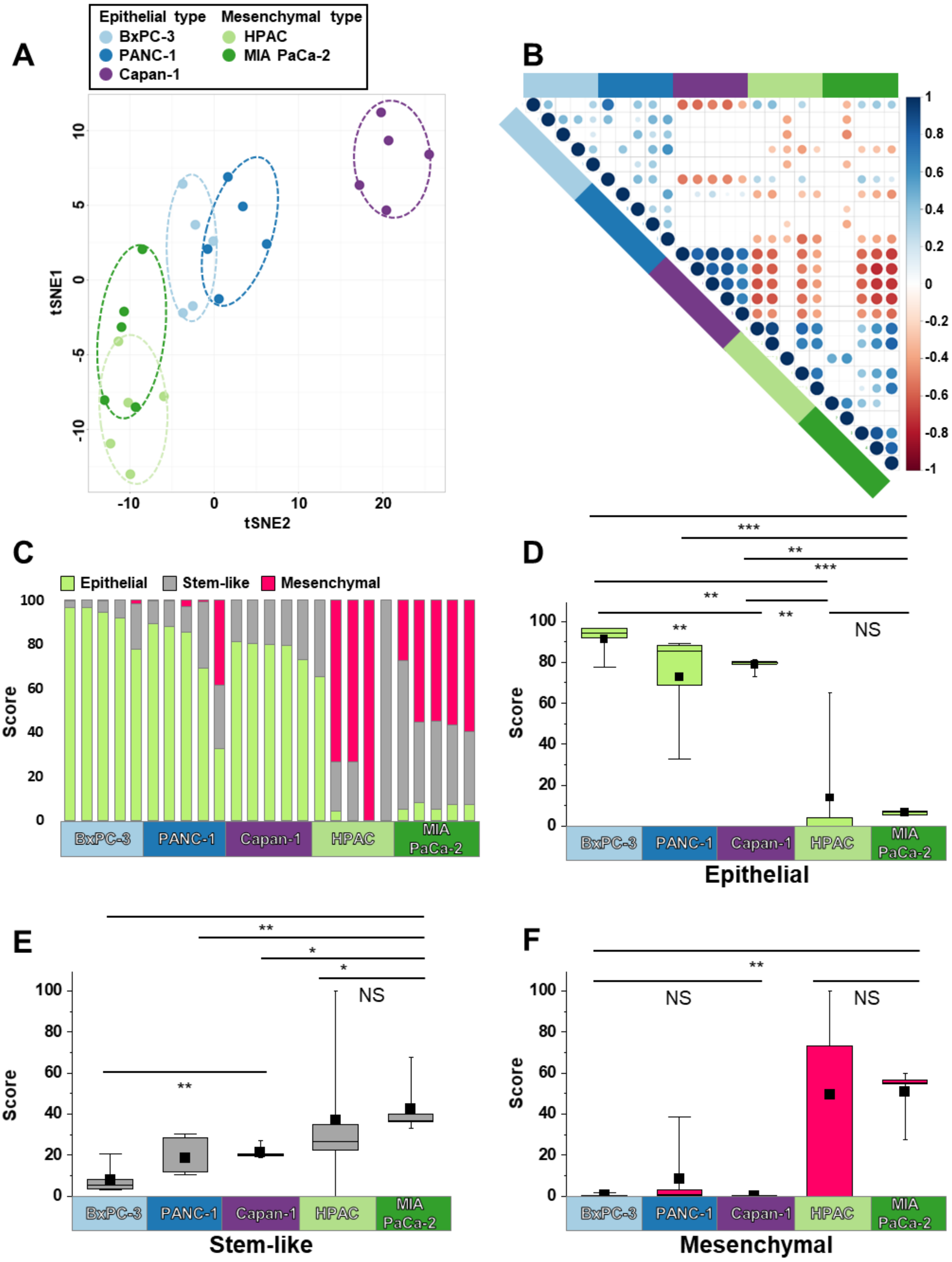

Figure S4. Characterization of pancreatic cancer cell lines using mRNA profiling. (**A**) 2D t-SNE analysis based on mRNA profiling of five pancreatic cancer cell lines. (**B**) The correlation matrix plot of five pancreatic cancer cell lines. High and low similarities are indicated with blue and red color based on the scale bar, respectively. The circle size represents the magnitude of the correlation. *p*-values in this correlation analysis were derived using the Cor.mtest function in R. The Bonferroni correction was applied to *p*-values to account for multiple testing in the rank correlation matrix. (**C**) Circulating tumor cell (CTC) classification in three categories (epithelial, stem-like, and mesenchymal CTCs) for five

pancreatic cancer cell lines. (**D**) The epithelial score, (E) stem-like score, and (F) mesenchymal score of five cell lines. Box plots show 25th and 75th percentiles with lines indicating the median value and black square dots indicating the mean value (*$p < 0.05$, **$p < 0.01$, ***$p < 0.001$, NS: non-significant). (Epithelial score: BxPC-3, 91.5 ± 7.0; PANC-1, 72.9 ± 21.4; Capan-1, 78.8 ± 2.9; HPAC, 13.9 ± 25.7; and MIA PaCa-2, 6.5 ± 1.2).

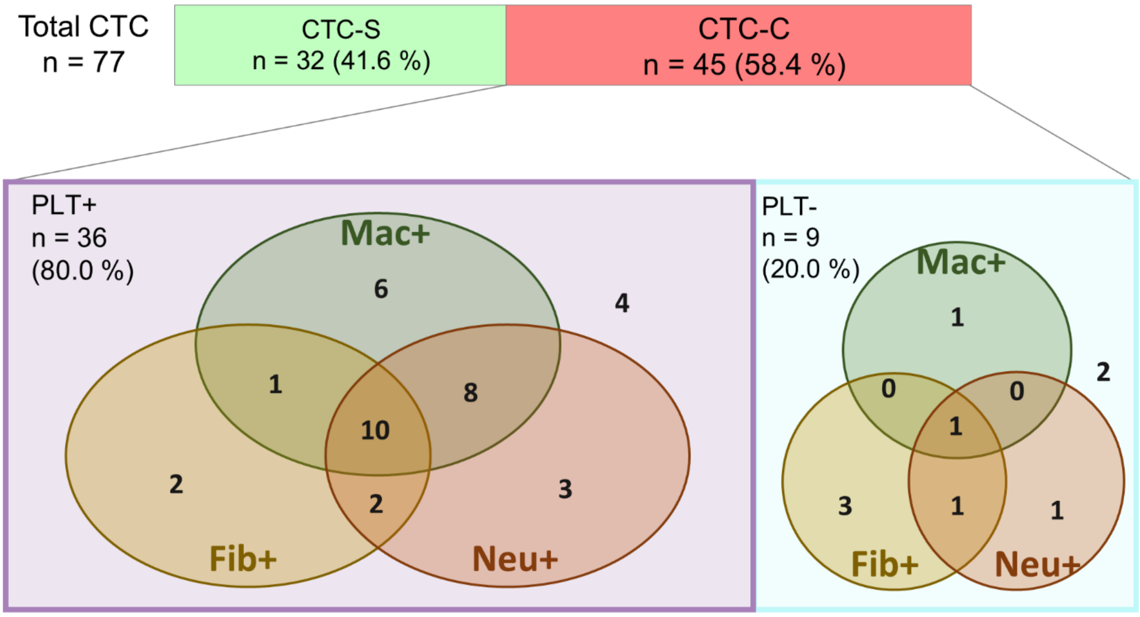

Figure S5. Investigation of heterogeneous components in circulating tumor cell (CTC) clusters. Venn diagram of CTC proportion according to the expression of platelets (PLT), macrophages (Mac), neutrophils (Neu), and fibroblasts (Fib). CTC-S, single CTC; CTC-C, CTC cluster.

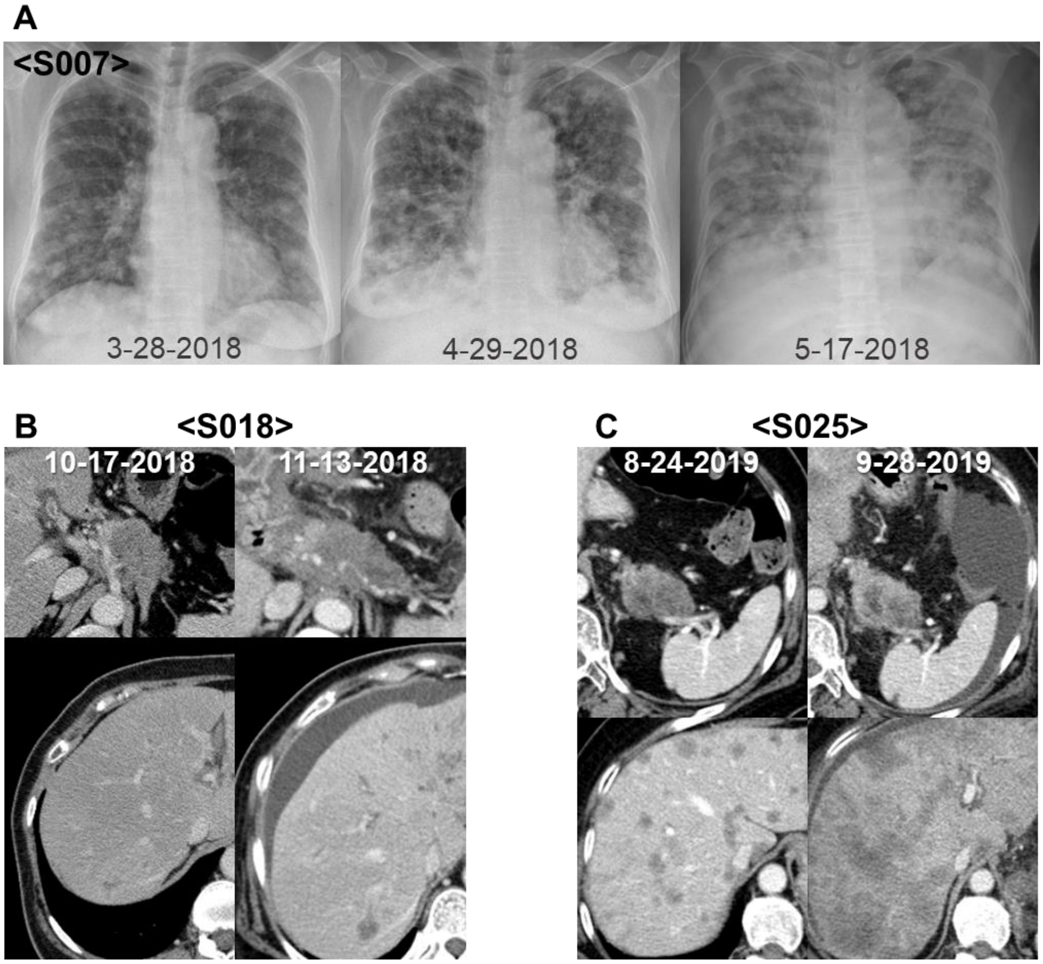

Figure S6. Serial chest X ray images and computed tomography (CT) images of patients in the rapid progression in metastasis (RP-M) group. (**A**) Patient S007 showed rapid progression of lung metastasis in serial chest X ray images. (**B**) Patient S018 showed increased primary pancreatic and hepatic masses and new appearance of ascites on the computed tomography image. (**C**) Patient S025 showed massive extension of hepatic mass and new appearance of ascites.

Table S1. Marker list.

Housekeeping gene	ACTb	Beta-actin
	GAPDH	Glyceraldehyde-3-phosphate dehydrogenase
Epithelial marker	CD276	B7-H3; immune check point molecule
	CEACAM5	Carcinoembryonic antigen-related cell adhesion molecule 5; carcinoembryonic antigen (CEA)
	E-cadherin	CDH1; E-cadherin; liver cell adhesion molecule (LCAM)
	EPCAM	Epithelial cellular adhesion molecule; tumor-associated calcium signal transducer (TACSTD1)
	KRT14	Kerain 14; cytokeratin 14
	KRT19	Keratin 19
	KRT7	Keratin 7
	MUC1	Mucin 1, transmembrane
	MUC2	Mucin 2
Mesenchymal marker	N-cadherin	CDH2; cadherin, neuronal
	SLUG	Snail family transcriptional repressor 2 (SNAI2)
	SNAIL	Snail family transcriptional repressor 1 (SNAI1)
	SPARC	Secreted protein acidic and rich in cysteine; Basement-membrane protein 40 (BM-40); Osteonectin
	TWIST1	Twist, drosophila, homolog of 1
	VIM	Vimentin
Stem cell-like marker	CD44	CD44 antigen
	NANOG	Nanog homeobox
	PROM1	Prominin-1; CD133
	CD24	CD24 antigen
	CD34	CD34 molecule
Platelet marker	PDGFb	Platelet-derived growth factor subunit B
	ITGA2b	Integrin alpha-IIb; CD41
	SELP	P-selectin, CD62P
Macrophage marker	ADGRE1	Adhesion G protein-coupled receptor E1; EGF-like module-containing mucin-like hormone receptor-like 1 (EMR1)
	CD14	Cluster of Differentiation:929

	CD68	Cluster of Differentiation 68
Neutrophil marker	CD45	PTPRC; protein-tryosine phosphatase, receptor-type, C; leukocyte-common antigen
	CSF3R	granulocyte colony-stimulating factor receptor (G-CSF-R); Cluster of Differentiation 114 (CD114)
	ITGAM	Integrin alpha M; Macrophage-1 antigen (Mac-1); Complement receptor 3 (CR3); Cluster of differentiation molecule 11B (CD11B)
Fibroblast marker	S100A4	S100 calcium-binding protein A4
	THY1	Thy-1 cell surface antigen; CD90
Drug response marker	ABCC1	Multidrug resistance-associated protein 1 (MRP1)
	ABCC3	Canalicular multispecific organic anion transporter 2 (cMOAT2)
	ERCC1	Excision repair cross-complementation group 1
	NFAT5	Nuclear factor of activated T-cells 5
	PRM1	Protamine 1
	TOP2A	DNA topoisomerase 2-alpha
	TYMS	Thymidylate synthase (TS)
Proliferative marker	CCNB1	Cyclin B1
	CCND1	Cyclin D1
	Ki67	MKI67, marker of proliferation Ki-67
Immunogenic marker	CD274	Programmed death-ligand 1 (PD-L1); B7 homolog 1 (B7-H1)
	PDCD1	Programmed cell death protein 1 (PD-1); CD279
Oncogene marker	EGFR	Epidermal growth factor receptor; HER1; ERBB1
	ERBB2	HER2; V-ERB-B2 avian erythroblastic leukemia viral oncogene homolog 2; NEU
	KRAS	K-ras, KRAS proto-oncogne

Table S2. Patient information.

Patient Code	Sex/ Age	Stage	Chemotherapy	CA19-9 (U/mL)	Follow up (days)	PFS (days)	OS (days)	Cause of Death	No. CTCs used for mRNA profiling							
									V0		V1		V2		V3	
									CTC-S	CTC-C	CTC-S	CTC-C	CTC-S	CTC-C	CTC-S	CTC-C
S007	F/66	M	GEM 1cycle	33775	51	51	51	PDAC	3	8	-	-	-	-	-	-
S010	F/61	M	GEM 6cycles	816	311	170	311	PDAC	0	1	0	0	0	0	1	4
S011	M/51	M	GEM 6cycles	1208	213	105	243	PDAC	0	0	-	-	-	-	-	-
S012	M/80	LA	GEM 6cycles	174	381	303	388	PDAC	2	0	0	0	0	0	-	-
S013	F/72	LA	FFX 5cycles	423	295	144	376	PDAC	0	0	0	0	-	-	-	-
S015	M/80	M	GEM 2cycles	7572	175	34	184	PDAC	0	1	-	-	-	-	-	-
S016	F/62	LA	GA 4cycles	783	286	114	291	PDAC	0	0	2	1	4	2	-	-
S018	F/73	M	GEM 1cycle	12320	52	38	52	PDAC	2	17	-	-	-	-	-	-
S019	M/74	LA	GEM 6cycles	2	347	267	347	Alive	0	0	0	0	-	-	-	-
S020	M/61	LA	FFX 10cycles	528	302	257	302	PDAC	13	2	1	0	-	-	-	-
S021	M/55	M	GA 5cylces	100000	186	171	186	PDAC	0	0	-	-	-	-	-	-
S022	F/63	M	FFX 4cycles	8	173	110	173	PDAC	0	0	-	-	-	-	-	-
S023	M/65	M	FFX 11cycles	704	320	268	320	Alive	0	0	-	-	-	-	-	-
S024	F/70	M	None	16	48	63	63	Sudden cardiac arrest	0	0	-	-	-	-	-	-
S025	F/64	M	None	8.16	45	45	45	PDAC	4	9	-	-	-	-	-	-
S026	F/74	M	FFX 11cycles	8	244	244	244	Alive	0	0	-	-	-	-	-	-

V0: baseline, M: metastatic disease, LA: locally advanced disease, GEM: Gemcitabine, FFX: FOLFIRINOX, GA: Gemcitabine + Abraxane, CTC-S: single CTC, CTC-C: CTC cluster, -: sample is not available (No sample collection)

Table S3. Circulating tumor cell (CTC) classification between single CTCs and CTC clusters

CTC type	Epithelial	Stem-like	Mesenchymal
CTC-S (*n* = 32)	46.50 ± 34.59	24.40 ± 23.55	29.10 ± 39.70
CTC-C (*n* = 45)	23.00 ± 33.37	13.40 ± 22.69	63.60 ± 39.35
p value	**0.004**	**0.046**	**0.0004**

Table S4. Circulating tumor cell (CTC) classification between single CTCs and CTC clusters according to the expression of platelet markers

CTC type	Epithelial	Stem-like	Mesenchymal
**PLT- (*n* = 27)**	**55.1 ± 37.0**	**23.7 ± 28.0**	**21.2 ± 36.9**
CTC-S (*n* =18)	55.8 ± 34.6	25.3 ± 23.7	18.8 ± 36.2
CTC-C (*n* = 9)	53.6 ± 45.5	20.5 ± 38.0	25.8 ± 42.1
**PLT+ (*n* = 50)**	**20.7 ± 28.7**	**14.9 ± 20.3**	**64.4 ± 38.2**
CTC-S (*n* =14)	34.5 ± 33.2	23.2 ± 25.1	42.2 ± 42.8
CTC-C (*n* =36)	15.3 ± 25.6	11.7 ± 17.8	73.0 ± 33.6
p value	0.00003	0.1632	0.00001

Table S5. Characterization of circulating tumor cell (CTC) clusters according to the stromal and immune cell marker expressions

CTC-C	# of PLT+ CTC-C	# of PLT- CTC-C
Mac+/Neu+/Fib+	10	1
Mac+/Neu+/Fib-	8	0
Mac+/Neu-/Fib+	1	0
Mac+/Neu-/Fib-	6	1
Mac-/Neu+/Fib+	2	1
Mac-/Neu+/Fib-	3	1
Mac-/Neu-/Fib+	2	3
Mac-/Neu-/Fib-	4	2
**Total**	**36 (80%)**	**9 (20%)**

PLT: platelet, Mac: macrophage, Neu: neutrophil, Fib: fibroblast

- Mac+/PLT+ CTC-C (*n* = 25) among Mac+ CTC-C (*n*=27) = 25/27 (92.6%)
- Neu+/PLT+ CTC-C (*n* = 23) among New+ CTC-C (*n*=26) = 23/26 (88.5%)
- Fib+/PLT+ CTC-C (*n* = 15) among Fib+ CTC-C (*n*=20) = 15/20 (75.0 %)

Table S6. Portion of the cases for the comparison between RP-M and Non-RP-M group

**Patient Group**	**CTC type**	**Epithelial (%)**	**Stem-like (%)**	**Mesenchymal (%)**	**PLT+ (*n*)**	**PLT- (*n*)**
**RP-M (*n* = 3)**	CTC-C (*n* = 34)	7.4	10.1	82.6	31	3
	CTC-S (*n* = 9)	11.3	23.2	65.4	8	1
Subtotal	*n* = 43	8.2	12.8	**79.0**	**39/43 (90.7%)**	**4/43 (9.3%)**
**Non-RP-M (*n* = 5)**	CTC-C (*n* = 11)	**71.1**	23.9	5.0	5	6
	CTC-S (*n* = 23)	60.3	24.9	14.9	6	17
Subtotal	*n* = 34	63.8	24.5	**11.7**	**11/34 (32.4%)**	**23/34 (67.6%)**
